# Exploring the relationship between anxiety, depression and wellbeing in doctors: a national cross-sectional survey and interviews

**DOI:** 10.1192/bjo.2021.85

**Published:** 2021-06-18

**Authors:** Emma Boxley, Gemma Simons, John Jenkins

**Affiliations:** 1 University of Southampton; 2 Centre for Workforce Wellbeing, University of southampton

## Abstract

**Aims:**

To examine the relationship between depression, anxiety and wellbeing in doctors.

**Background:**

The relationship between doctor wellbeing and mental health diagnoses is not well evidenced in the literature. There is a lack of comparable measurement of wellbeing in doctors within the National Health Service, meaning the effectiveness of wellbeing interventions is unknown.

**Method:**

A cross-sectional survey containing the PHQ9, GAD7 and WEMWBS questionnaires to measure depression, anxiety and wellbeing respectively, was advertised online nationally. The relationships between the total scores were explored using Spearman's rho correlation coefficients and Chi square tests. Thematic analysis of semi-structured interviews offered further insights.

**Result:**

Sixty-seven doctors returned completed questionnaires. 29.9% had PHQ9 scores >5 and 41.8% had GAD7 scores >5. Therefore, over a quarter of the participants had a score that would suggest a management plan was needed for depression, and a third for anxiety. Moderate negative correlation between the total WEMWBS scores and the total PHQ , rs= –0.775, p = 0.00, N = 67 and GAD7 scores rs= –0.724, , p = 0.00, N = 67 was seen. Statistically significant differences between those with low wellbeing scores (WEMWBS < 40) and normal wellbeing scores (WEMWBS ≥ 40) in relation to the need for a management plan for depression (PHQ9 > 10) X2 (1, N = 67) = 12.395, p = 0.00 and anxiety (GAD7>10) X2 (1, N = 67) = 5.611, p = 0.018 were seen. The main themes identified from the interviews (n = 10) were the importance of social support outside of work, cynicism about an NHS plan check-in and a tendancy to neglect wellbeing until it has dipped.

**Conclusion:**

There is a moderate negative correlation between anxiety, depression and wellbeing, but they are not opposites and separate measures for wellbeing should be used. It is clinically useful to note that only those with a WEMWBS score of <45 had a PHQ9 score suggesting the need for treatment of depression.

